# Navigating Complex Anatomy During Leadless Pacemaker Implantation

**DOI:** 10.1016/j.jaccas.2023.101912

**Published:** 2023-06-12

**Authors:** Lolita Golemi, Niteesh Chitturu, Hiren Patel, Yongzhen Chen, Ahmed Hussein

**Affiliations:** Saint Louis University School of Medicine, Department of Internal Medicine, St. Louis, Missouri, USA

**Keywords:** complex anatomy, hypoplasia, inferior vena cava filter, inferior vena cava malformations, scoliosis, leadless pacemaker, venous stenosis

## Abstract

Transvenous pacemakers may lead to wound site complications, such as hematomas and infections. Leadless pacemakers have eliminated these risks. However, when the central venous and/or cardiac anatomy are challenging, their implantation technique may require modification(s). Here, we discuss 3 cases of successful leadless pacemaker implantation in patients with a challenging anatomy. (**Level of Difficulty: Advanced.**)

## Central Illustration

**Figure:**
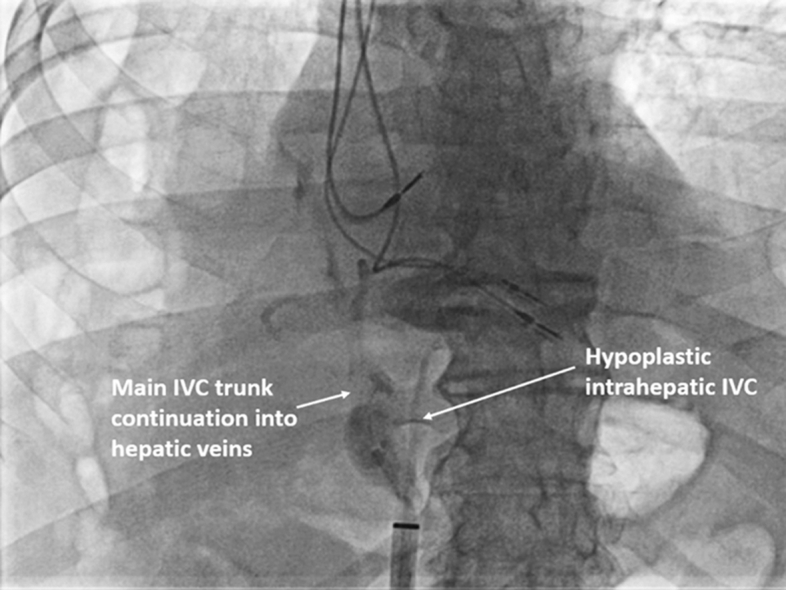

More than 1 million pacemakers are implanted every year.^1^ Transvenous pacing systems have been associated with several complications, including hematoma, lead dislodgement, infection, and lead perforation.^2^ With the recent increase in use of leadless pacemakers (LP), many of these risks have been eliminated.^1^ So far, little is known about the best approach to implant LP in patients with difficult anatomy. In this case series, we discuss novel modifications in the technique of LP implantation in 3 different patients with challenging central venous anatomy.

**Learning Objectives**

• To discuss the need for variations in the leadless pacemaker implantation technique in patients with complex anatomy including patients with inferior vena cava hypoplasia, inferior vena cava filters, and lumbar scoliosis.
• To understand the challenges and the possible need for modifications in the leadless pacemaker implantation technique in patients with end-stage renal disease patients on hemodialysis.

## Patient 1

**Figure 1:**
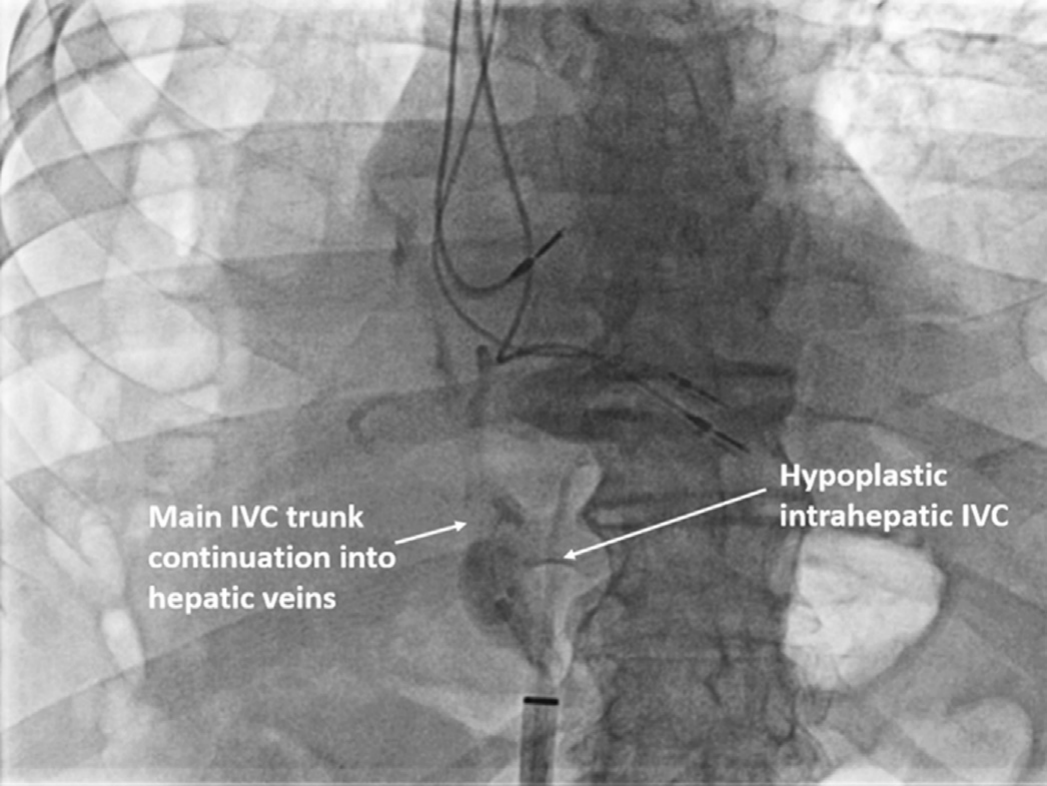
IVC Hypoplasia Fluoroscopic anterior posterior view after injection of contrast through the leadless pacemaker sheath in the inferior vena cava (IVC) demonstrating the IVC bifurcating into a medial hypoplastic intrahepatic IVC and lateral short wide trunk that gives rise to left, right, and middle hepatic veins.

A 68-year-old man was referred for extraction of infected pacemaker leads and LP implantation. During implantation, attempts to advance the wire through the inferior vena cava (IVC) were unsuccessful as the wire deviated to the hepatic veins. The LP introducer sheath (IS) was guided to the region of deviation, and a venogram was obtained, which showed selective injection in the hepatic veins with the right hepatic vein draining in the IVC (Video 1). The IS was withdrawn to a lower level in the IVC, and another contrast injection demonstrated the IVC bifurcating into a medial hypoplastic segment and a lateral shorter and wider continuation further bifurcating into right and left hepatic veins, with the right hepatic vein reconnecting to the IVC at a slightly higher level (Figure 1, Video 2). A glidewire was used to cross the hypoplastic intrahepatic IVC segment to the superior vena cava (SVC). The IS dilator assembly was advanced over the glidewire with the leading dilator, dilating the hypoplastic IVC segment while meeting moderate resistance, until the IS could reach the right atrial lower border. The LP delivery catheter was then advanced through the tricuspid valve and implanted in right ventricular midseptal position.

## Patient 2

**Figure 2:**
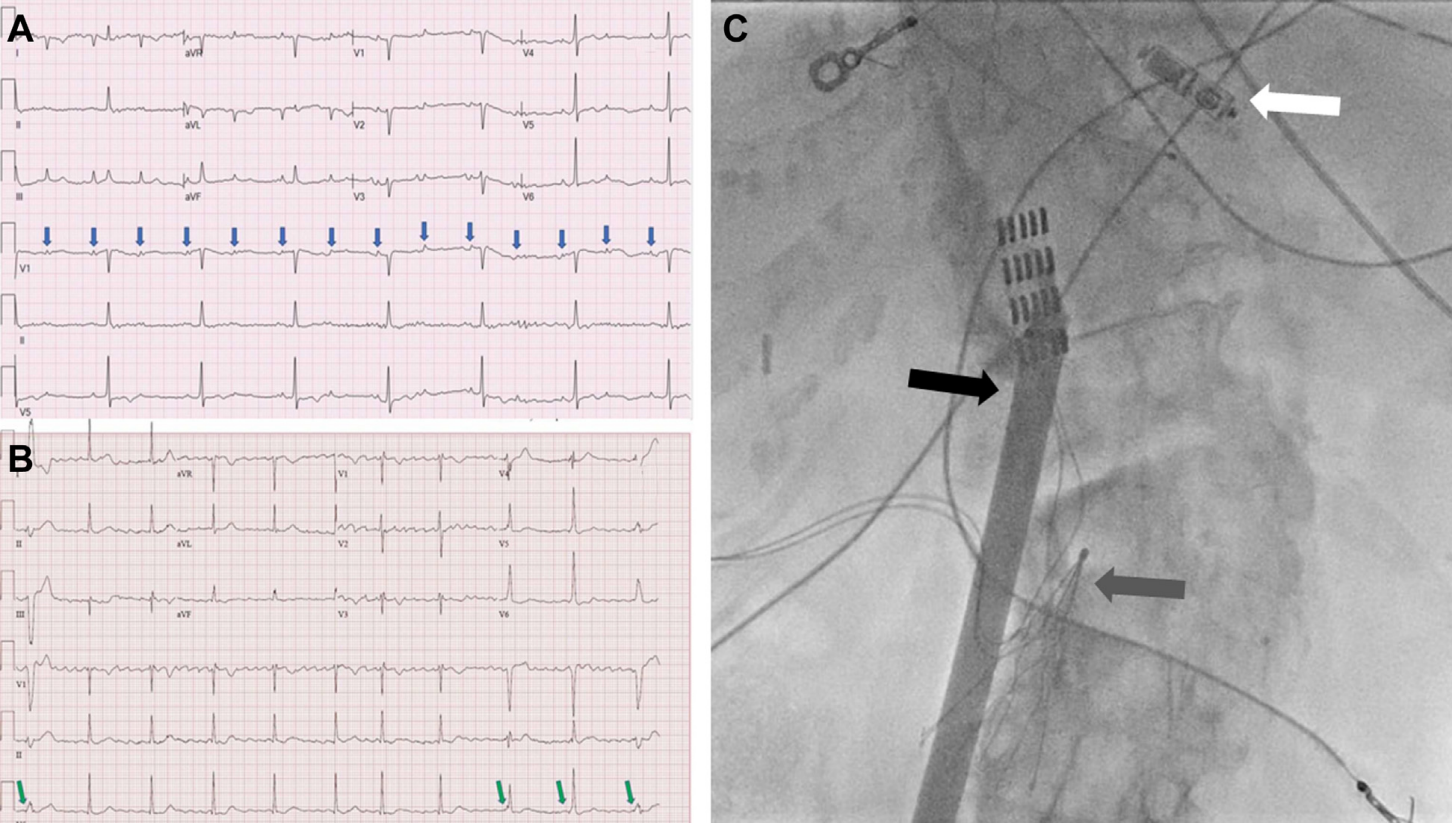
Pre- and Post-Leadless Pacemaker Implantation ECGs and a Postimplantation Fluoroscopic View Demonstrating the Venous Anatomical Difficulties Encountered **(A)** Preprocedural electrocardiogram (ECG) showing complete heart block. **(B)** Postprocedural ECG showing atrial fibrillation and ventricular pacing with adequate capture. **(C)** Anatomically tortuous course of the inferior vena cava (IVC) with lumbar spine scoliosis as demonstrated by contrast injection through the sheath, IVC filter **(grey arrow)**, angulated introducer sheath **(black arrow)** and a successfully implanted leadless pacemaker **(white arrow)**. A spine stimulator array of electrodes arranged in 4 rows appears near the top of the figure.

An 86-year-old woman presented with a left femur fracture sustained after a fall, which was attributed to pauses caused by intermittent complete heart block. An LP was offered. A preprocedural radiography demonstrated the presence of an intrabdominal IVC filter and severe lumbar scoliosis (Figure 2). A glidewire was used to cross the IVC filter and allow for greater maneuverability to pass struts of the filter and advance along the IVC distortion caused by scoliosis. A glide catheter was then passed over the wire to the SVC, and the glidewire was then replaced with an Amplatz stiff wire. The glide catheter was then removed leaving the stiff wire in the SVC. The IS dilator assembly was then advanced over the stiff wire to the lower right atrium (Videos 3A and 3B). Through the sheath, the LP delivery catheter was advanced to the right atrium, then through the tricuspid valve and implanted in right ventricular mid septal position. The IS was withdrawn under fluoroscopy to avoid IVC filter disruption (Video 4).

## Patient 3

**Figure 3:**
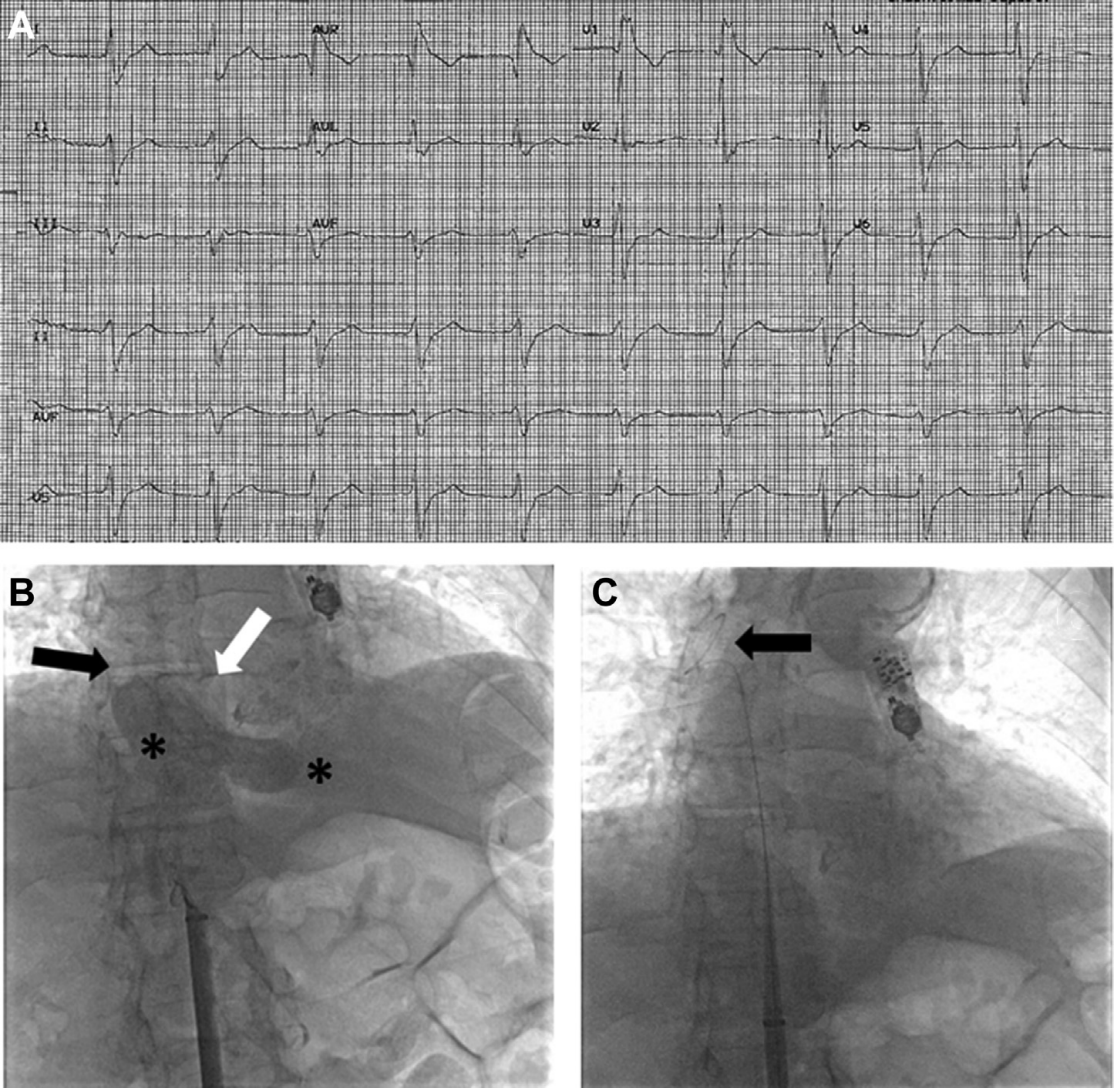
Pre-Leadless Pacemaker Implantation Electrocardiogram and Procedural Fluoroscopic Views Demonstrating the Venous and Cardiac Structural Anatomical Difficulties Encountered **(A)** A 12-lead electrocardiogram showing junctional rhythm with left anterior fascicular block and right bundle branch block. **(B)** Venogram with contrast injection at the intrahepatic inferior vena cava level showed superior vena cava (SVC) stenosis **(black arrow)** with severely enlarged left atrium **(white arrow)** and deformed elongated right atrium **(black asterisk)** with inferiorly displaced tricuspid valve **(white asterisk)**. The fluoroscopy frame was taken during expiration. **(C)** Posteroanterior fluoroscopic view demonstrating the ability to pass the guidewire beyond the SVC. The guidewire stops at the right brachiocephalic vein **(black arrow)**. A loop recorder appears near the top of the figure.

An 87-year-old man with a history of severe aortic stenosis, end-stage renal disease receiving hemodialysis via the left forearm arteriovenous fistula, right brachiocephalic vein (stenosis related to a prior indwelling dialysis catheter and eventually required balloon dilatation, presented to hospital with persistent dizziness. An electrocardiogram demonstrated sinus bradycardia in the 30s, first-degree atrioventricular block, and bifascicular block. He was offered an LP because of the history of central venous occlusion and to avoid the potential risk of transvenous pacing system infection being a hemodialysis patient.^3^ While attempting to advance the LP delivery catheter through the tricuspid valve, it kept entering the coronary sinus (Video 5). Therefore, we decided to study the right atrial and venous anatomy. The LP delivery catheter was retracted, and the IS was pulled to the IVC. A wire advanced through the IS could be passed up through the SVC to the level of the right brachiocephalic vein. However, injection of a small amount of contrast through the IS demonstrated restricted flow in the SVC (Video 6). That process was followed by a larger contrast injection that again demonstrated the restricted flow in the SVC, in addition to a distorted and elongated right atrium with anteriorly and inferiorly displaced tricuspid valve likely as a result of a severely enlarged left atrium (Figure 3, Video 7). Owing to these anatomic variations, the LP delivery catheter was pulled to a slightly lower level in the right atrium, and a significant counterclockwise rotation was applied to the catheter while pushing it anteriorly and inferiorly. These maneuvers eventually enabled the catheter to cross the tricuspid valve and to be advanced deeper in the right ventricle. However, with the angulations in the course of the catheter, it could only reach the apical septal region of the interventricular septum, rather than the usual mid septal position.

In all 3 cases, adequate pacing and sensing parameters were obtained and no complications occurred.

## Discussion

LPs are now considered the preferred alternatives to the traditional transvenous pacing systems in hemodialysis patients and patients at high risk of infection.^3^ To our knowledge, case 1 is the first reported case of implantation of LP in a patient with IVC hypoplasia. In that case, a glidewire, which is a softer and easier to maneuver, was used to cross the hypoplastic IVC segment, and the sheath dilator complex were carefully used to dilate that segment.

IVC filters have been considered a contraindication for LP placement, but several successful LP implantations in patients with IVC filters have been previously reported.^4^, ^5^, ^6^ A softer guidewire was typically used to pass through the widest part of the IVC filter, then exchanged for a stiffer wire for successful placement. In case 2, we successfully used a similar technique to cross the IVC filter, despite the added complexity conferred by a significantly tortuous IVC because of severe scoliosis.

Central veins stenosis has been reported to occur in 25% to 47% of patients with end-stage renal disease. Although central veins stenosis may alter the anatomical course needed for LP implantation, they are preferred over conventional transvenous pacing systems to avoid further exacerbation of the stenosis. In case 3, the patient had history of right brachiocephalic stenosis that seemed to have progressed to SVC stenosis, which may have contributed to the deformation of the right atrium and displacement of the tricuspid valve in the presence of a severely enlarged left atrium. Successful LP implantation was achieved only after applying a significant counterclockwise rotation to the LP delivery system to be able to cross the anteriorly displaced tricuspid valve.

## Conclusions

With the increasing use of LPs, it is expected that more patients with challenging anatomy will be encountered. In these patients, implantation of LPs is safe and feasible. However, additional tools, implantation technique modifications, and a careful review of additional imaging may be needed for successful implantation.

## Funding Support and Author Disclosures

The authors have reported that they have no relationships relevant to the contents of this paper to disclose.

## Appendix

Video 1 Fluoroscopic Run in Anteroposterior View with First Contrast Injection through the Leadless Pacemaker Introducer Sheath The contrast delineated the hepatic veins that appear to be in direct continuation with the infrahepatic inferior vena cava. The right hepatic vein could be seen draining into the inferior vena cava.

Video 2 Fluoroscopic Run in Anteroposterior View with Second Contrast Injection through the Leadless Pacemaker Introducer Sheath The contrast demonstrated the inferior vena cava bifurcating into a medial hypoplastic segment and a lateral shorter and wider continuation further bifurcating into right and left hepatic veins.

Video 3A AFluoroscopic Run in Anteroposterior View Demonstrating Sheath–dilator Assembly Advancement and the Sheath Final Position Before Passing the Leadless Pacemaker Delivery Catheter through It **(A)** The introducer–sheath assembly was carefully advanced over the Amplatz wire. Significant angulation of the dilator tip can be seen as the sheath–dilator assembly advanced along the markedly scoliotic spine. **(B)** Notice the significant curvature of the sheath and its passage through the inferior vena cava filter. Incidentally, a previously implanted spinal stimulator can be seen in this video.

Video 3B BFluoroscopic Run in Anteroposterior View Demonstrating Sheath–dilator Assembly Advancement and the Sheath Final Position Before Passing the Leadless Pacemaker Delivery Catheter through It **(A)** The introducer–sheath assembly was carefully advanced over the Amplatz wire. Significant angulation of the dilator tip can be seen as the sheath–dilator assembly advanced along the markedly scoliotic spine. **(B)** Notice the significant curvature of the sheath and its passage through the inferior vena cava filter. Incidentally, a previously implanted spinal stimulator can be seen in this video.

Video 4 Fluoroscopic Run in Anteroposterior Demonstrating Sheath Withdrawal under Fluoroscopy The sheath was withdrawn under fluoroscopy to avoid inferior vena cava (IVC) filter disruption. A small amount of contrast was continuously injected to delineate the significant IVC deformation caused by the lumbar scoliosis.

Video 5 Fluoroscopic Run in the Right Anterior Oblique View Demonstrating Leadless Pacemaker Delivery Catheter Entering a Narrow Tubular Structure Injection of contrast through the catheter delineated a portion of the coronary sinus that is located inferior to the calcified mitral valve.

Video 6 Fluoroscopic Run in Anteroposterior Demonstrating the Stiff Wire Advancement Tthrough the SVC Although the wire could be advanced, through the inferior vena cava (SVC), the injection of a small amount of contrast demonstrated restricted flow of contrast in the SVC.

Video 7 Fluoroscopic Run in Anteroposterior Demonstrating Contrast Injection in the IVC to Identify the Anatomical Variations A greater amount of contrast was injected in the inferior vena cava (IVC) and that demonstrated restricted flow in the superior vena cava suggestive of significant stenosis, in addition to a distorted elongated right atrium with anteriorly and inferiorly displaced tricuspid valve, likely as a result of a severely enlarged left atrium.
